# Comparing locomotor intensity indicators in soccer training and competition across contextual factors: a study of replaced coaches in a Portuguese professional 1st league team

**DOI:** 10.3389/fspor.2024.1391784

**Published:** 2024-05-24

**Authors:** Honorato Sousa, Filipe Manuel Clemente, Hugo Sarmento, Élvio R. Gouveia, Rabiu Muazu Musa

**Affiliations:** ^1^ Research Unit for Sport and Physical Activity, Faculty of Sport Sciences and Physical Education, University of Coimbra, Coimbra, Portugal; ^2^ Escola Superior Desporto e Lazer, Instituto Politécnico de Viana do Castelo, Rua Escola Industrial e Comercial de Nun’Álvares, Viana do Castelo, Portugal; ^3^ Sport Physical Activity and Health Research & Innovation Center, Viana do Castelo, Portugal; ^4^ Gdansk University of Physical Education and Sport, Gdańsk, Poland; ^5^ LARSyS, Interactive Technologies Institute, Funchal, Portugal; ^6^ Department of Physical Education and Sport, University of Madeira, Funchal, Portugal; ^7^ Centre for Fundamental and Continuing Education, Universiti Malaysia Terengganu, Kuala Nerus, Malaysia

**Keywords:** manager, football, changes, contextual variables, physical effects

## Abstract

This study aims to examine, for each head coach (HC) replaced, the association between training intensity and physical performances obtained in games. Furthermore, the study investigated how contextual factors influence locomotor and mechanical performance association. External load variables were collected using Global Positioning System (GPS) devices across the 4 weeks and 4 games before and after the replacement in a professional adult male soccer team. Six different HC records were analysed (48.8 ± 7.4 years of age; 11.2 ± 3.9 years as an HC) during a three-season span (2020/21–2022/2023). There were marked differences within player variability across the two coaching regimes. Game loads didn't reflect training-related performance, with differences ranging from −71.4% to −9.9%. Players under the outgoing coaches have greater coverage of meters per minute. Meters per minute, distance covered over 18 km/h and high-speed running (all in training) are found to be significant variables influenced by contextual factors. Within-subject and time, training loads did not reflect game-related loads/performances, with starters showing higher deficits (ranging from −79.0 to −14.5). The study suggests that changes in soccer HC can affect players' training intensity and game performance, influenced by various contextual factors and not directly correlated. This type of information might be very suitable to improve training load periodization and programming. For further research avenues, could be the study of the variation of the psychological states of the players at the time of the dismissal and hiring of the HCs, associating them with the physiological performance at the same moments.

## 1 Introduction

Over the years, training control programs and teams' preparation processes started to find a need to control technical and physical elements in an isolated way, intending to predict performance in competition (1, 2). With player's technical and physical responses being determined by tactical behaviours in competition, competitive demands are influenced by contextual variables (3–6). Despite tactical, technical, physiological and even logistical factors affecting professional soccer players' performance, nowadays, the press, the fans and the club's board management stare at winning as the most important aspect of professional soccer; assessing a team's success mainly through victory or defeat (7). During the season, in the presence of failure, the tendency is to replace the head coach (HC) with a new one, even with all the substantial disturbances in player and team stability that this situation can cause (8). Yet, current research supports that to achieve victory, players must acquire a high level of psychological and physical conditioning, besides the appropriate technical-tactical preparation (9), also showing that success in professional soccer is complex and multifactorial (3) and that HC has a fundamental part on the coach-athlete-performance process (8, 10).

Research directed towards the influence of contextual factors showed that there are physiological and technical effects on players' performance in professional soccer (11–13). It has also been identified that performance metrics, such as external load variables, can vary according to certain match factors, given that soccer is associated with various and complex logistical dynamics (14–17). Also, a set of contextual factors have been researched for their ability to constrain the performance demands of soccer match-play. Among those situational factors, the quality of the opponent (better/worst/equal position in the league's table), the match location (away or home game), and the match status (if the team wins, draws or loses in a competitive game), are the most prevalent reported in the scientific community (14–19).

In line with the above, information about the physiological, locomotor and mechanical impact of competition on male professional soccer players can assume a significant role in managing the recovery strategies regarding the impact of these demands on the players (19). For that, GPS devices have become an indispensable tool to quantify the player's load and support coaches in their ability to study the dynamics of the load during training sessions and matches, providing relevant information in each scenario (2, 11). Consequently, meticulously managing both overall workload and specific weekly training loads has emerged as a crucial strategy for inducing physiological adaptations in professional soccer players. This enhanced outcome is achieved through their skilled optimization of performance, which accelerates recovery, diminishes injury risk, and ensures greater player availability (20–23). Furthermore, it enables the head coach and team staff to tailor training interventions to meet the specific demands of upcoming games that the team and players will encounter (19, 24).

Despite this evidence, research on how contextual factors affect players' physiological performance is limited. Moreover, data is sparse regarding the impact of an HC's mid-season entry or exit on team and player performance and the wider consequences prompted by these contextual changes. To the best of the authors' knowledge, no research has focused on understanding what a change of HC, associated with an analysis of context variables, can promote in terms of physiological effects on the players and in their preparation for competition. Grasping how various contextual factors, both logistical and competitive, influence physical loads is essential for optimizing training, fostering beneficial physiological changes, and enhancing performance.

Therefore, this study aimed to (1) analyse for each HC status whether there is an association between training intensity and performances obtained in the game and (2) examine the degree of association in the indicators of locomotor and mechanical performance between training and competition, depending on contextual factors: (i) player's performance in game and training locomotive variables under different coach regimes; (ii) match location (home or away); (iii) opponent's position in the table; (iv) game result; (v) starters and reserves players' performances in-game and training locomotive variables.

## 2 Material and methods

### 2.1 Participants

During this three-season span, a total of 6 different HCS were at the club (48.8 ± 7.4 years of age; 11.2 ± 3.9 years as a head coach; all with UEFA Pro License). In the 1st season, a total of three HCs; in the 2nd season, the HC that finished the 1st season started but did not finish, summing 2 HCs; in 3rd season, the HC that ended the 2nd season started but did not end, with the team having two more HCs till the end of the season. All dismissals happened for motives linked to poor results and lowly performances. All relevant performance indicators of this club's competitive record during the three-season period under analysis are in the public domain. Sports-specialized platforms, such as zerozero.pt and transfermarkt.com, provided the data. The information related to the date of signing and dismissal of coaches and match results, and the game sheets that showed the starting 11 and playing time of each player that participated on the games, was consistent in both sources, validating the data presented. The extraction of this information was carried by one author (HS) with a user account in both platforms to have access to all data available. Each procedure applied was approved by the Ethics Committee of the University of Coimbra, reference: CE/FCDEF-UC/00042023. The investigation was conducted following the Declaration of Helsinki.

### 2.2 Study design and setting

This cross-sectional observational study is part of another published study (25). The data collection occurred in all training sessions and official matches of a professional adult male soccer club during three complete sporting seasons (2020/21, 2021/22 and 2022/2023). To perform the analyses on the relations of locomotor intensity indicators between training and game contexts, when also comparing contexts between coaches, the 4 weeks and 4 games before the replacement of the old coach were analysed, as well as the first 4 weeks and 4 games after the arrival of the new coach. The sample size was determined based on the number of games and training sessions over three consecutive seasons. Consequently, *a priori* sample size analysis was conducted using G*Power to determine the sample size required to draw meaningful conclusions. The 4 weeks (about 20 training sessions) demonstrated that 12 training sessions (effect size and *P* defined at 0.40 and 0.05, respectively) will allow to generate a statistical effect significant (power of 0.90). Therefore, a sample size of 20 games and all training sessions from the 4-week span selected were deemed sufficient for this study to avoid the problem of Type II error (26, 27). In this framework, representation is attained by the number of training sessions/games, not by the number of players. The rationale of using 12 games and training sessions as the minimum requirement seems to be a practical decision based on the structure of the sports season and the need to have a sufficient number of observations for each player. In a typical sports season, there are numerous training sessions, and choosing a minimum of 12 ensures that the data collected is representative of the player's performance throughout the season. It also allows for the analysis of trends and patterns over time (28, 29). In Sousa et al. (25), the aim was to forecast the retention or dismissal of coaches by checking the physical effects of mid-season replacements and investigating which external load and physical performance variables in training and competition can predict retention or dismissal. This research focuses on evaluating the impact of contextual factors on the external load experienced by players in training and competition, also incorporating the HC as a covariate. This data drifted from the same dataset as the first study, but there were changes in the way the data was analysed. For reliability and validation of the study, only data from players who participated in the full sessions' duration have been analysed, removing the data from the goalkeepers (from all sessions and games), players whose training load was influenced due to fatigue management (total of 21 individual data reports removed), players who were returning from injury (total of 10 individual data reports removed) and players who were injured during the training sessions (total of 7 individual data reports removed. Concerning the data from the games, only information was included on the group of players who participated in the games, whether as starters or substitutes. Also, data from training sessions immediately after the day of the game, with no day off between, where the team was divided into two different working groups with two different training plans (starters and reserve players groups), was not considered (total of 3 training sessions removed).

### 2.3 Instruments

The external load variables were collected using two Global Positioning System (GPS) devices (1st season—10-Hz GPS unit EVO, Catapult; 2nd and 3rd seasons—STATSports APEX, 10 Hz augmented GNSS). To wear these devices in a suitable manner, a skin-tight bag was used in the thoracic region between the scapulae. The players used the same devices (individual identification on the equipment) in training and official games from a league with 34 fixtures. The technical team was responsible for the placement and use of the equipment (GPS and Radar), and there may be the possibility of some misplacement of the devices. Situations regarding the life battery of the devices ending during a session were not part of the data, as they did not include the total duration of the training session.

### 2.4 Variables under study

The study utilized external load variables to quantify the volume and intensity of physical activity. Regarding volume: (1) the total distance covered during games (TD-G) and training sessions (TD-T) reflects overall exercise volume and an individual's contribution to team efforts in total meters; (2) distances traversed at speeds above 14 km/h (zone 4), 18 km/h (zone 5), and 24 km/h (zone 6) were recorded separately for games (DB4-G, DB5-G, DB6-G) and training (DB4-T, DB5-T, DB6-T), providing detailed speed-specific distance data; (3) high-speed running, indicating distances covered at speeds greater than 18 km/h and 24 km/h, was tracked for games (HSR-G) and training (HSR-T) and presented in total meters; (4) acceleration instances in games (AC-G) and training (AC-T) were counted when players exceeded a threshold of 3 m/s² for at least one second, presented in the form of frequency of occurrences (*n*=); (5) similarly, deceleration counts were noted for games (DC-G) and training (DC-T), when deceleration surpassed 3 m/s² for the same duration, also presented by the frequency of occurrences.

About the intensity variables: (1) maximum speed in the game (MS-G) and training (MS-T), measuring top speed as the maximum speed prolonged for at least half a second (km/h); (2) meters per minute in-game (MM-G) and training (MM-T), reporting the complete distance in meters covered, divided by the duration of the session/exercise, presented by the number of meters completed during one minute (MM).

An internal load training intensity control variable was also added, specifically the rate of perceived exertion (RPE-T), namely the Foster and colleagues validated scale (30). Every time a training session ended, players individually, so that they would not be influenced by the opinions of other players, indicated the amount of individual effort undertaken during action. A trainer assisted with this action using a tablet device showing the validated scale. The player had to select a number on a scale of 1–10, with 1 being the lowest value and 10 being the maximal effort.

### 2.5 Data treatment and statistical analysis

The data collected in this study were subjected to a comprehensive statistical analysis. To avoid any potential biases arising from differing units of measurement, the data were normalized. Given the nature of the data, which includes measurements from forty games and training sessions each week for three consecutive seasons, we employed a Mixed-Effects Model. This model is particularly suitable for repeated measures data, which is the case here as we have multiple measurements (games and training sessions) for the same subjects (players).

The assumptions for this model include the Independence of observations: This is addressed by the study design, where each game and training session is considered an independent event. Normal distribution of residuals: This was verified using a Q-Q plot. Homogeneity of variance: This was checked using Levene's test. The selection of this model is justified as it allows for the inclusion of both fixed effects (such as coaching regimes and player status as starters or reserves) and random effects (such as individual player differences). It also accounts for the correlation between repeated measures on the same subjects.

In addition to the Mixed-Effects Model, we also conducted descriptive statistics to summarize the overall locomotive performance of the team. This includes measures of central tendency (mean) and dispersion (standard deviation). We classified the coaching period as either an entry or an exit period based on the first four or the last four games of the coach, respectively. This classification was done to study the effect of coaching transition on the team's performance. The Chi-Square Test of Independence was used to examine the relationship between the coaching period and game outcomes.

Finally, we studied various game variables, such as venue, final game results, and opponent strength. These variables were included in the model as covariates. The selection of these covariates was based on their potential influence on the team's locomotive performance. The significance of these covariates was tested using Wald's test. This comprehensive statistical approach allows us to understand the trade-offs between games and training loads, the impact of different coaching regimes, and the performance differences between starters and reserve players. It also provides insights into the effects of various game variables on the team's performance.

The statistical analysis in this study involved several tests and assumptions. While the Shapiro-Wilks test confirmed the normal distribution of the sample, however, it is known to be sensitive to large sample sizes. Hence, the Box's test checked the sphericity assumption, indicating equal variances of differences between all related groups, and found no violation (*p* > 0.05). The 2-way repeated measures ANOVA with a between and within-subjects design was used to ascertain the differences between game and training locomotive variables based on the status of the coaches i.e., exit or entry and players' roles (starters and reserve players). The independence of observations within each sample, an inherent assumption in ANOVA, was also met. It is worth highlighting that despite the inherent limitations of ANOVA that consist of being sensitive to outliers and not robust to violations of the sphericity assumption as well as complex to interpret, especially if there is a three-way interaction or higher, its application in the current study is deemed appropriate due to the ability to meet the assumptions of the test. Additionally, the Chi-Square Test for Goodness of Fit and the Kruskal Wallis Test were used, each with their own assumptions and limitations. For instance, the Chi-Square test assumes that observations are independent and that the sample size is sufficiently large, while the Kruskal Wallis test assumes that samples are independent and identically shaped. Moreover, Cohen's d effect size analysis was employed to examine the magnitude of differences within the study sample which was evaluated using the Hopkins scale as follows: 0–0.2, trivial; 0.2–0.6, small; 0.6–1.2, moderate; 1.2–2.0, large; >2.0, very large (31, 32). The Eta Squared for the (ANOVA) was interpreted as η^2^ < 0.01, negligible, small; 0.01 ≤ η^2^ < 0.06, medium; 0.06 ≤ η^2^ < 0.14; large: η^2^ ≥ 0.14 (33). For the chi-square test, a value of Phi (φ) = 0.1 is considered to be a small effect, 0.3 a medium effect, and 0.5 a large effect (34). All inferences were set at an alpha (α) level of ≤0.05 using the SPSS statistical software package (SPSS Inc., Chicago, IL, USA, 20.0) and XL STAT add-in software version 2014 for Windows.

## 3 Results

**Figure 1:**
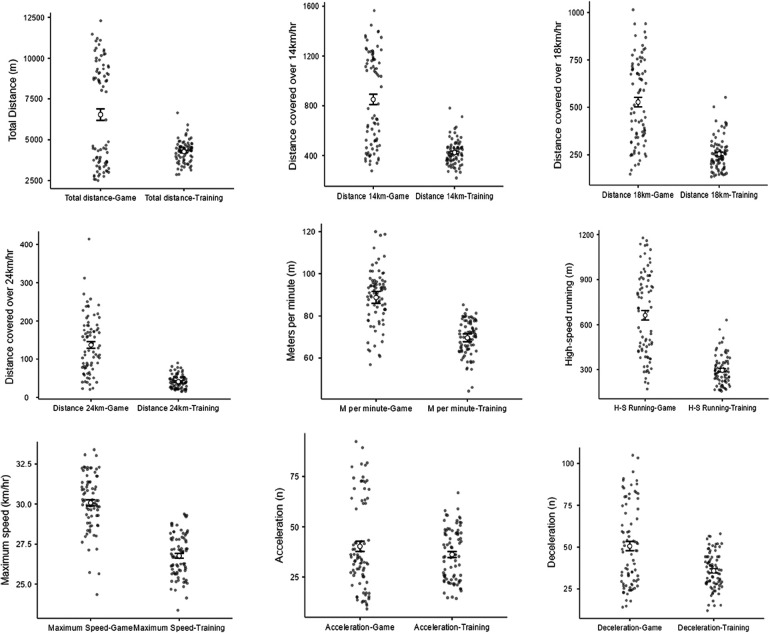
Significant difference in player variation between game and training loads.

Table 1 reflects the differences in the team performances based on the games and training locomotive variables based on coaches' status (i.e., entry or exit). Significant interactions were found for within-subject and time (game vs. training) in 8 of the 9 measured variables (the exception was accelerations). There are marked differences within player variability across all the two coaching regimes in which game loads do not reflect training-related performance, with differences ranging from −71.4 to −9.9%. Distance-related coverage and high-speed running were observed to be highly unbalanced across the two coaches. Figure 1 depicts the trade-off between game and training for within-player variability.

**Table 1:** Analysis of player’s performance in game and training locomotive variables under different coach regimes.

Variables	Coach Status	Game (Mean ± SD)	Microcycle training (Mean ± SD)	% Difference	Within group (Player variation)	Between Group (game)	Between group (Training)
Total Distance (m)	Entry	6,438.5 ± 3,126.78	4,224.8 ± 712.14	−34.4	*F* = 35.98; *p* < 0.001**; η²*p* = 0.316	*t* = −0.280; *p* = 0.780; *d* = 0.0626	*t* = 0.676; *p* = 0.501; *d* = 0.151
	Exit	6,635.5 ± 3,169.15	4,325.6 ± 616.56	−34.8			
Meters per Minute (m)	Entry	85.3 ± 12.96	69.8 ± 7.96	−18.2	*F* = 5.87; *p* < 0.001**; η²*p* = 0.070	*t* = 2.469; *p* = 0.016*; *d* = −0.552	*t* = −0.225; *p* = 0.822; *d* = −0.050
	Exit	92.3 ± 12.40	69.3 ± 8.90	−24.9			
Distance covered over 14 km/h (m)	Entry	817.6 ± 380.96	430.9 ± 114.44	−47.3	*F* = 0774; *p* < 0.001**; η²*p* = 0.544	*t* = 0.777; *p* = 0.439; *d* = −0.174	*t* = −0.584; *p* = 0.561; *d* = −0.131
	Exit	882.4 ± 364.29	418.0 ± 79.95	−52.6			
Distance covered over 18 km/h (m)	Entry	508.3 ± 229.20	268.9 ± 85.19	−47.1	*F* = 96.68; *p* < 0.001**; η²*p* = 0.553	*t* = 0.746; *p* = 0.458; *d* = −0.167	*t* = −1.589; *p* = 0.116; *d* = - 0.355
	Exit	545.8 ± 219.72	238 ± 83.54	−56.4			
Distance covered over 24 km/h (m)	Entry	138.7 ± 84.71	43.8 ± 20.20	−68.4	*F* = 114.77; *p* < 0.001**; η²*p* = 0.595	*t* = −0.225; *p* = 0.822; *d* = −0.050	*t* = −1.348; *p* = 0.181; *d* = −0.301
	Exit	134.9 ± 66.00	38.6 ± 14.08	−71.4			
High-Speed Running (m)	Entry	644.9 ± 284.88	313.3 ± 96.76	−51.4	*F* = 112.27; *p* < 0.001**; η²*p* = 0.590	*t* = 0.564; *p* = 0.575; *d* = 0.126	*t* = −1.659; *p* = 0.101; *d* = −0.371
	Exit	680.5 ± 280.33	277.9 ± 94.31	−59.2			
Maximum Speed (km/h)	Entry	29.9 ± 1.96	26.9 ± 1.51	−10.0	*F* = 209.99; *p* < 0.001**; η²*p* = 0.729	*t* = 1.189; *p* = 0.238; *d* = 0.266	*t* = −0.502; *p* = 0.617; *d* = −0.112
	Exit	30.3 ± 1.38	26.7 ± 1.14	−11.9			
Accelerations (n)	Entry	41.4 ± 24.59	37.3 ± 15.20	−9.9	*F* = 3.064; *p* = 0.084; η²*p* = 0.038	*t* = −0.678; *p* = 0.500; *d* = −0.151	*t* = 0.307; *p* = 0.760; *d* = 0.068
	Exit	39.2 ± 20.45	35.3 ± 10.82	−9.9			
Decelerations (n)	Entry	49.8 ± 26.52	36.7 ± 12.65	−26.3	*F* = 24.66; *p *< 0.001**; η²*p* = 0.240	*t* = 0.307; *p* = 0.760; *d* = 0.068	*t* = 0.805; *p* = 0.423; *d* = −0.180
	Exit	51.5 ± 22.92	34.7 ± 8.98	−32.6			

* Significant difference over time (*p* < 0.05)

** Significant different within player variation.

**Figure 2:**
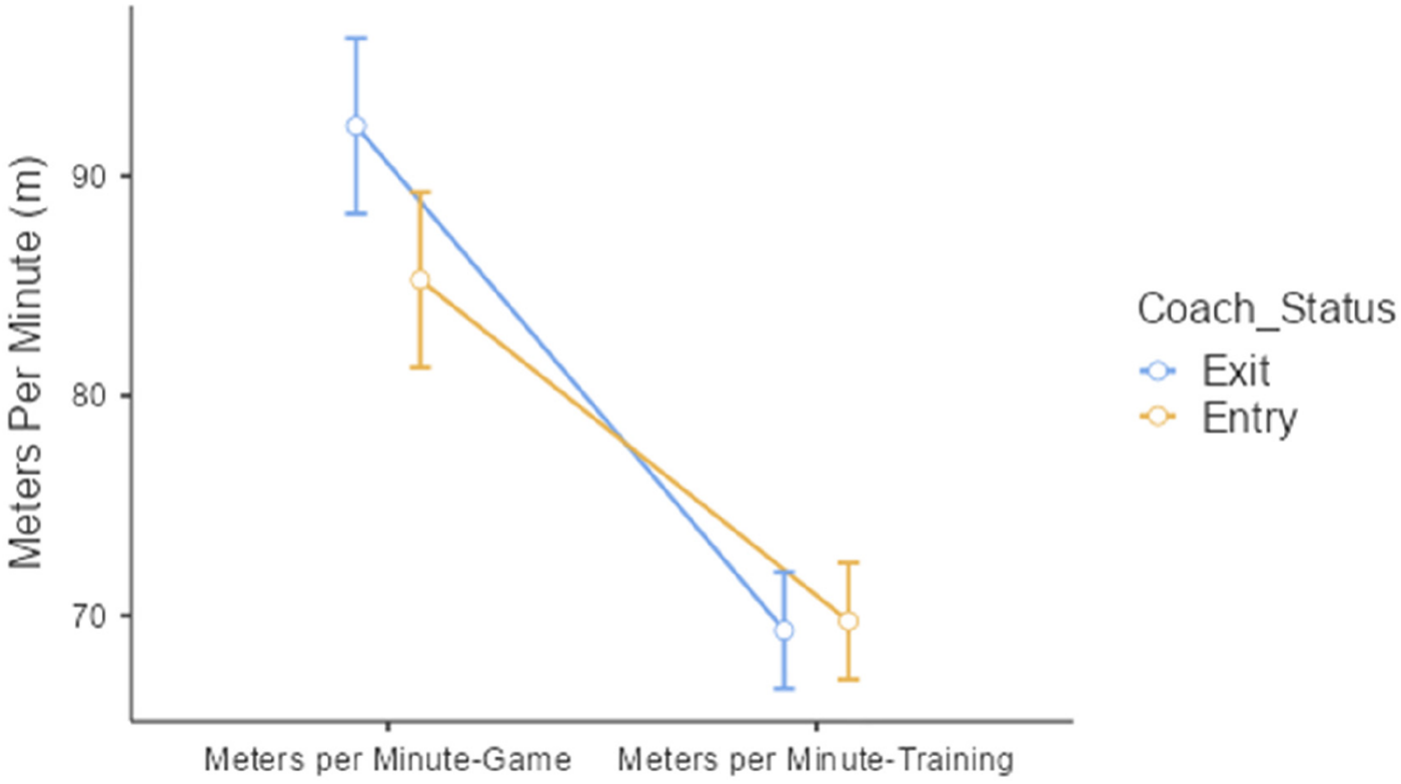
Significant locomotive variation between the exit and entry coach (relative total distance).

Comparison between subjects on game and training locomotive variables revealed a significant difference in meters per minute during the game. As illustrated in Figure 2, players under the outgoing coaches (exit) have greater coverage of meters per minute compared with those under the incoming coaches (entry) (*p* < 0.05). No other significant difference was observed in other variables between-subject comparisons.

**Figure 3:**
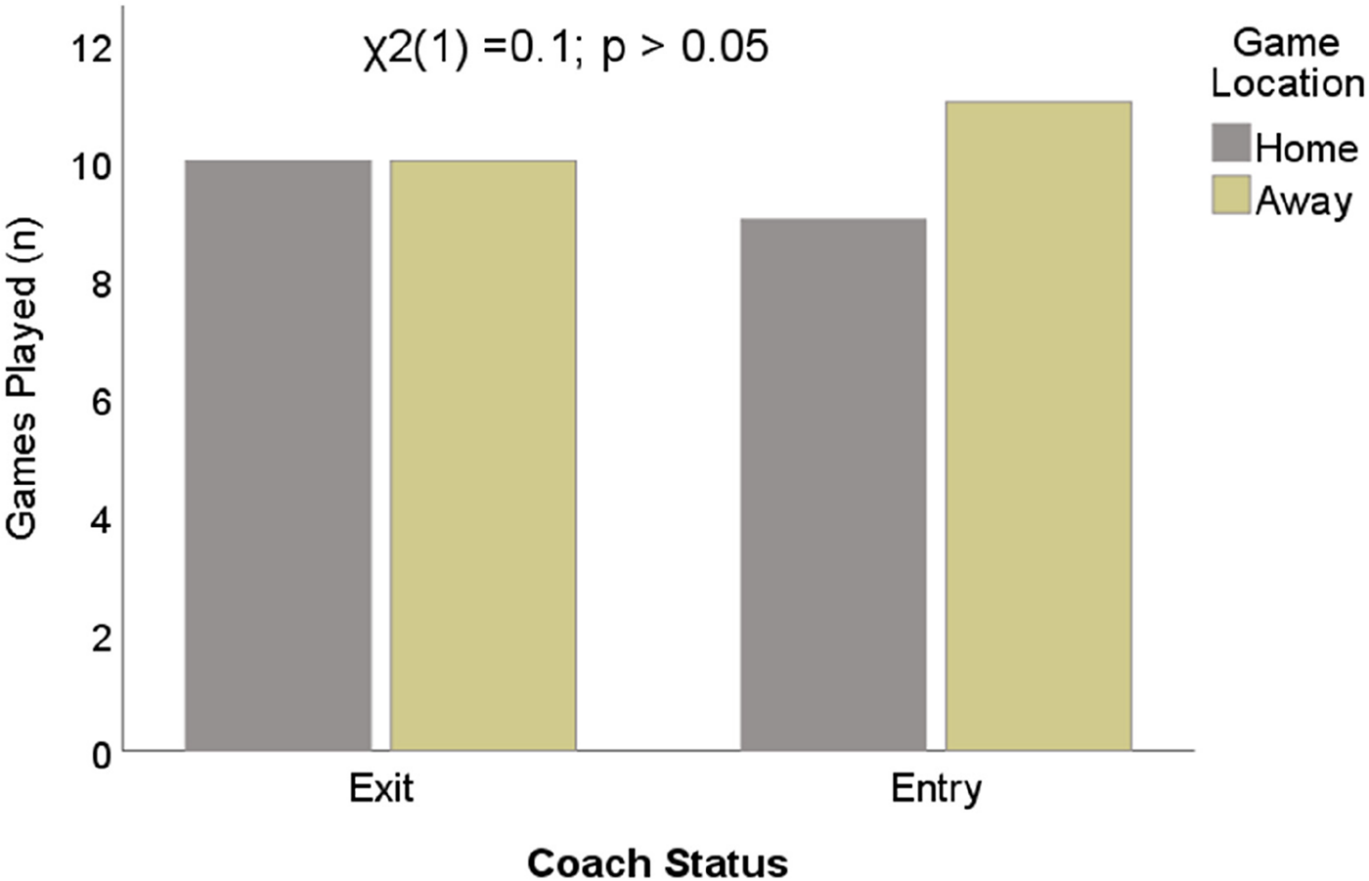
Differences in games played based on locations.

Figure 3 shows the Chi-square analysis of the differences between coaches' status with reference to the locations of the game. No statistically significant differences were observed between the variables. Hence, the games played between the coaches are fairly distributed across the two different locations i.e., home, and away.

**Figure 4:**
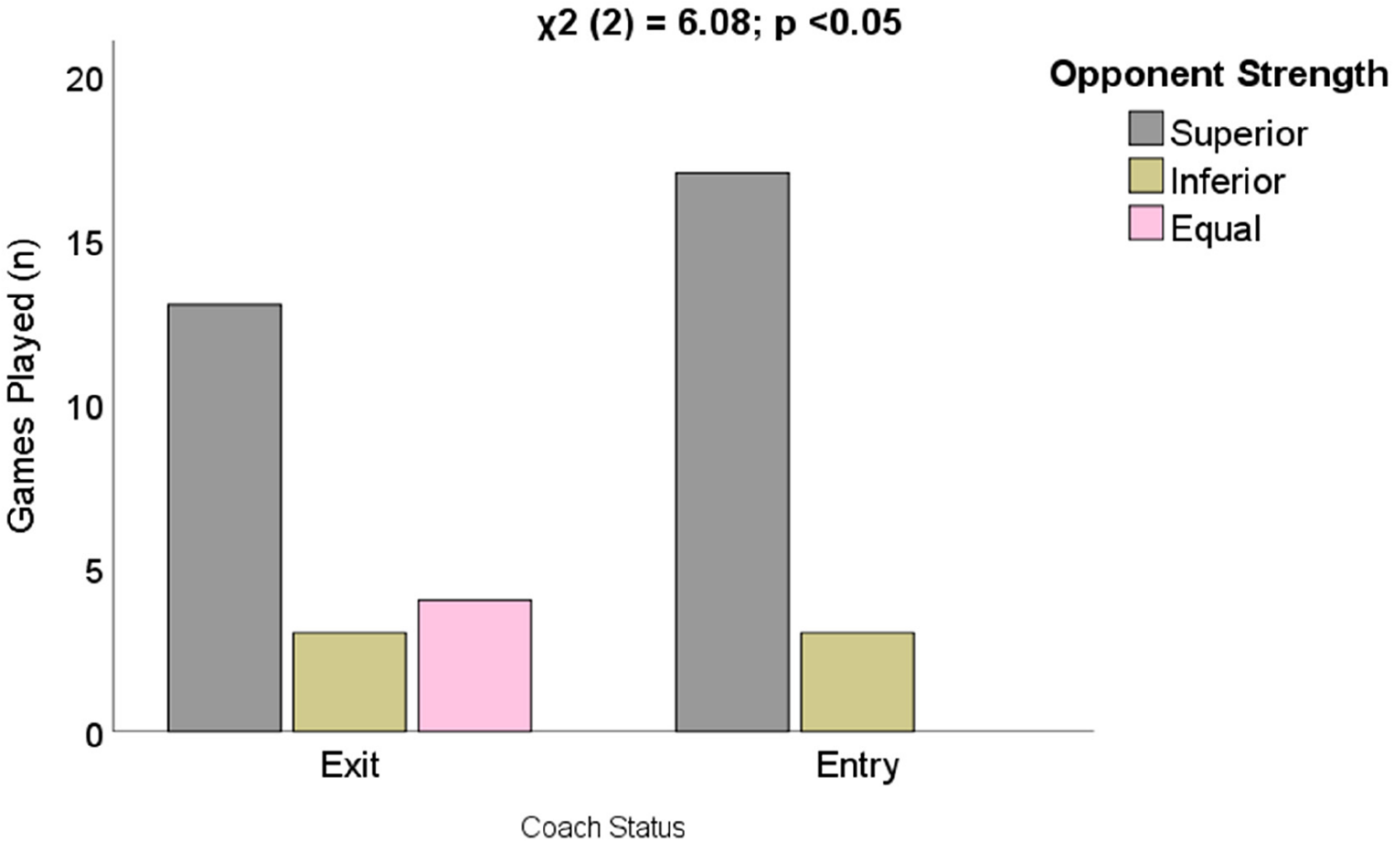
Differences in games played based on the opponent's strength.

**Figure 5:**
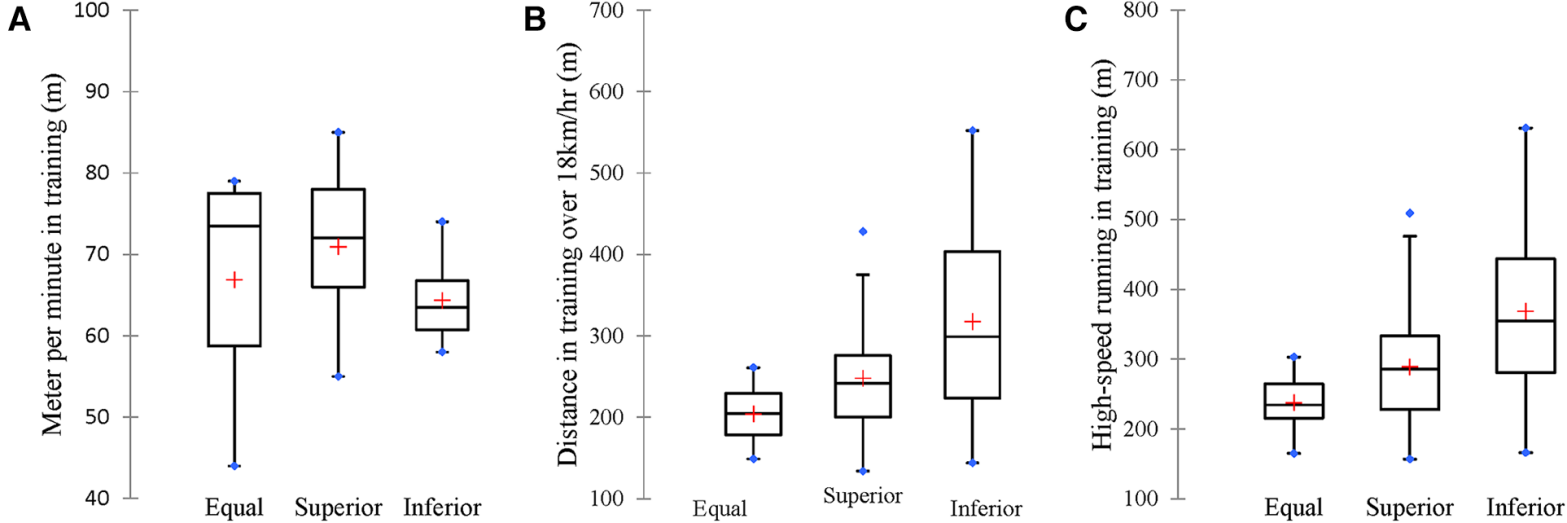
Significant locomotive variables (**A–C**) differentiating the team when playing against opponents of different strengths.

Figure 4 depicts the Chi-square analysis of the differences between the coaches' status regarding the strength of the opposition teams. Statistically significant differences were observed between the variables. The coaches who were fired had played with the superior team 13 times, 4 times with a team of equal strength, while the incoming coaches had played against the superior team 17 times, did not play with a team of equal strength, and both coaches have played with a team of inferior strength. Hence, it could be deduced that the incoming coaches (entry) have faced tougher challenges with respect to the opposition compared to the exited coaches. To further elucidate the link between the status of coaches and the performance of the players in the game and training locomotive variables, we followed up this analysis with the Kruskal Wallis test. The results from the Kruskal Wallis test revealed that only 3 training locomotive variables are significant. These variables, including meters per minute in training, distance covered over 18 km/hr in training and high-speed running in training, are found to be significant variables that differentiate the performance of the team under the two coaches' status. The results are depicted in Figures 5A–5C, in which the meters per minute performance of the team when playing with the superior team was higher; on the other hand, distance coverage of 18 km/h, as well as high-speed running, were found to be higher when playing with the inferior team. These findings reflect the importance of training loads in the performance of the players during match day.

**Figure 6:**
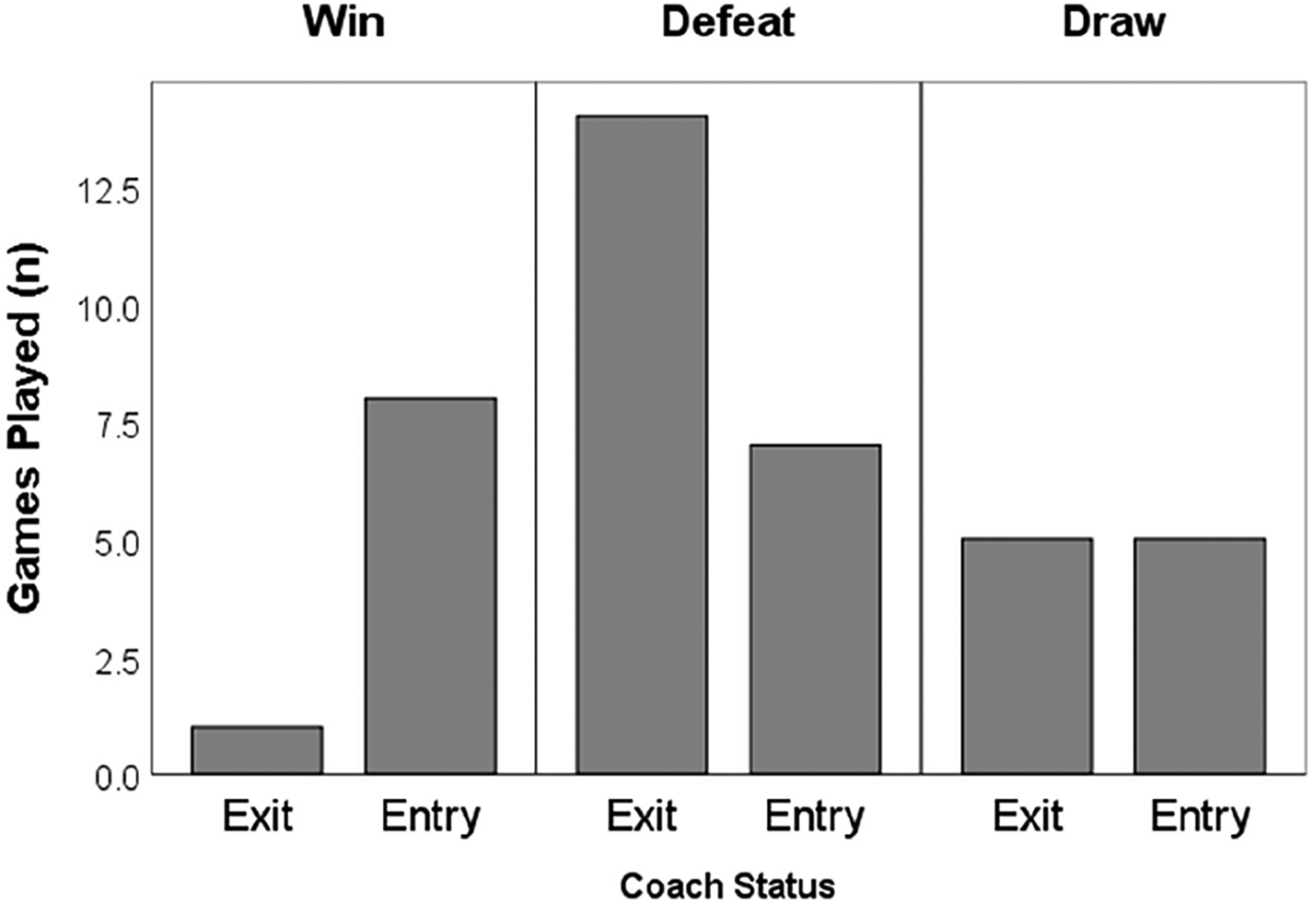
Differences in game outcomes between the two coaches.

Figure 6 displays the Chi-square analysis of the differences between the coach's status with reference to the match outcome. Statistically significant differences were observed between coaches based on the games' results. There were 40 games analysed across all three seasons, corresponding to the analysis period prior to dismissals and the analysis period immediately after arrivals. Amid coaches' status, each group managed 20 games in total. It is worth noting that the outcome of the game's results between coaches differs. The new coaches (entry) recorded 8 wins, 5 draws, and 7 defeats, while the coaches who were fired (exit) recorded 1 win, 5 draws, and 14 defeats. Based on these differences, we applied a Kruskal Wallis test to determine the difference between game locomotives and the game outcome during each coaching period. No statistically significant differences were observed with respect to the performance of the game and training locomotive variables when the team was winning, losing, or drawing across the two coaching regimes.

Table 2 reflects the differences in the team performances based on the games and training locomotive variables based on starters and reserve players. Significant interactions were found for within-subject and time (game vs. training) across all the measured variables. Marked differences in player variability across all the locomotive variables reveal that training loads do not reflect game-related loads/performances. Surprisingly, starters were found to have higher deficits of performance between game and training loads, with differences ranging from −79.0% to −14.5%. None of the locomotive variables are found to be balanced across starters, while reserve players demonstrate an increase in performance of total distance accelerations as well as decelerations. These findings revealed that reserve players are more likely to perform better in training as opposed to the starters, suggesting that the intensity and volume of training loads during the week, combined with factors such as specific strategies and preparation for the starting eleven for the next game, can influence the total load of players considering their status.

**Table 2:** Analysis of starters and reserves players’ performances in game and training locomotive variables.

Variables	Players’ roles	Game (*n* = 40)	Microcycle training (*n* = 40)	% Difference	Within group (Player variation)	Between group (Game)	Between group (Training)
Total Distance (m)	Starters	9,503.0 ± 1,162.98	4,107.9 ± 681.05	−56.8	*F* = 601; *p* < 0.001***; η²*p* = 0.885	*t* = −28.01; *p* < 0.001**; *d* = −0.626	*t* = 2.31; *p* = 0.023*; *d* = 0.518
	Reserves	3,570.9 ± 664.76	4,442.4 ± 609.34	+24.4			
Meters per Minute (m)	Starters	93.7 ± 6.65	68.8 ± 8.35	−26.6	*F* = 15; *p* < 0.001***; η²*p* = 0.161	*t* = −3.593; *p* < 0.001**; *d* = −0.−8,031	*t* = −0.784; *p* = 0.435; *d* = −0.175
	Reserves	83.9 ± 15.91	70.3 ± 8.48	−16.2			
Distance covered over 14 km/ (m)	Starters	1,179.1 ± 178.37	408.1 ± 107.44	−65.4	*F* = 272; *p *< 0.001***; η²*p* = 0.777	*t* = −17.31; *p* < 0.001**; *d* = −0.−872	*t* = −1.50; *p* = 0.138; *d* = 0.335
	Reserves	520.9 ± 161.22	440.8 ± 86.51	−15.4			
Distance covered over 18 km/h (m)	Starters	720.5 ± 127.21	237.8 ± 91.79	−67.0	*F* = 197; *p* < 0.001***; η²*p* = 0.716	*t* = −15.57; *p* < 0.001**; *d* = −0.−481	*t* = −1.71; *p* = 0.092; *d* = 0.382
	Reserves	333.5 ± 92.43	269.9 ± 75.77	−19.1			
Distance covered over 24 km/hr (m)	Starters	180.4 ± 49.07	37.8 ± 16.84	−79.0	*F* = 43.1; *p* < 0.001***; η²*p* = 0.356	*t* = −6.32; *p* < 0.001**; *d* = −0.413	*t* = 1.75; *p* = 0.085; *d* = −0.391
	Reserves	93.2 ± 72.28	44.6 ± 17.71	−52.1			
High-Speed Running (m)	Starters	895.7 ± 162.43	276.1 ± 103.03	−69.2	*F* = 160; *p* < 0.001***; η²*p* = 0.672	*t* = −13.31; *p* < 0.001**; *d* = −0.977	*t* = −1.84; *p* = 0.070; *d* = 0.411
	Reserves	429.7 ± 150.46	315.1 ± 86.2	−26.7			
Maximum Speed (km/h)	Starters	31.0 ± 1.03	26.5 ± 1.43	−14.5	*F* = 47.4; *p* < 0.001***; η²*p* = 0.378	*t* = −6.02; *p* < 0.001**; *d* = −0.846	*t* = −2.06; *p* = 0.043*; *d* = −0.460
	Reserves	29.1 ± 1.71	27.1 ± 1.16	−6.9			
Accelerations (n)	Starters	55.8 ± 20.63	34.2 ± 13.10	−38.7	*F* = 225.8; *p* < 0.001***; η²*p* = 0.743	*t* = −8.48; *p* < 0.001**; *d* = −0.897	*t* = −1.42; *p* = 0.159; *d* = −0.318
	Reserves	24.8 ± 10.38	38.4 ± 13.03	+54.8			
Decelerations (n)	Starters	71.6 ± 16.39	32.5 ± 10.81	−54.6	*F* = 374; *p* < 0.001***; η²*p* = 0.827	*t *= −14.66; *p* < 0.001**; *d* = −0.967	*t* = 2.68; *p* = 0.009*; *d* = −0.600
	Reserves	29.6 ± 7.68	38.9 ± 10.25	+31.4			

* Significant difference over time (*p* < 0.05).

** Significant difference over time (*p* < 0.001).

*** Significant differences within player variation.

**Figure 7:**
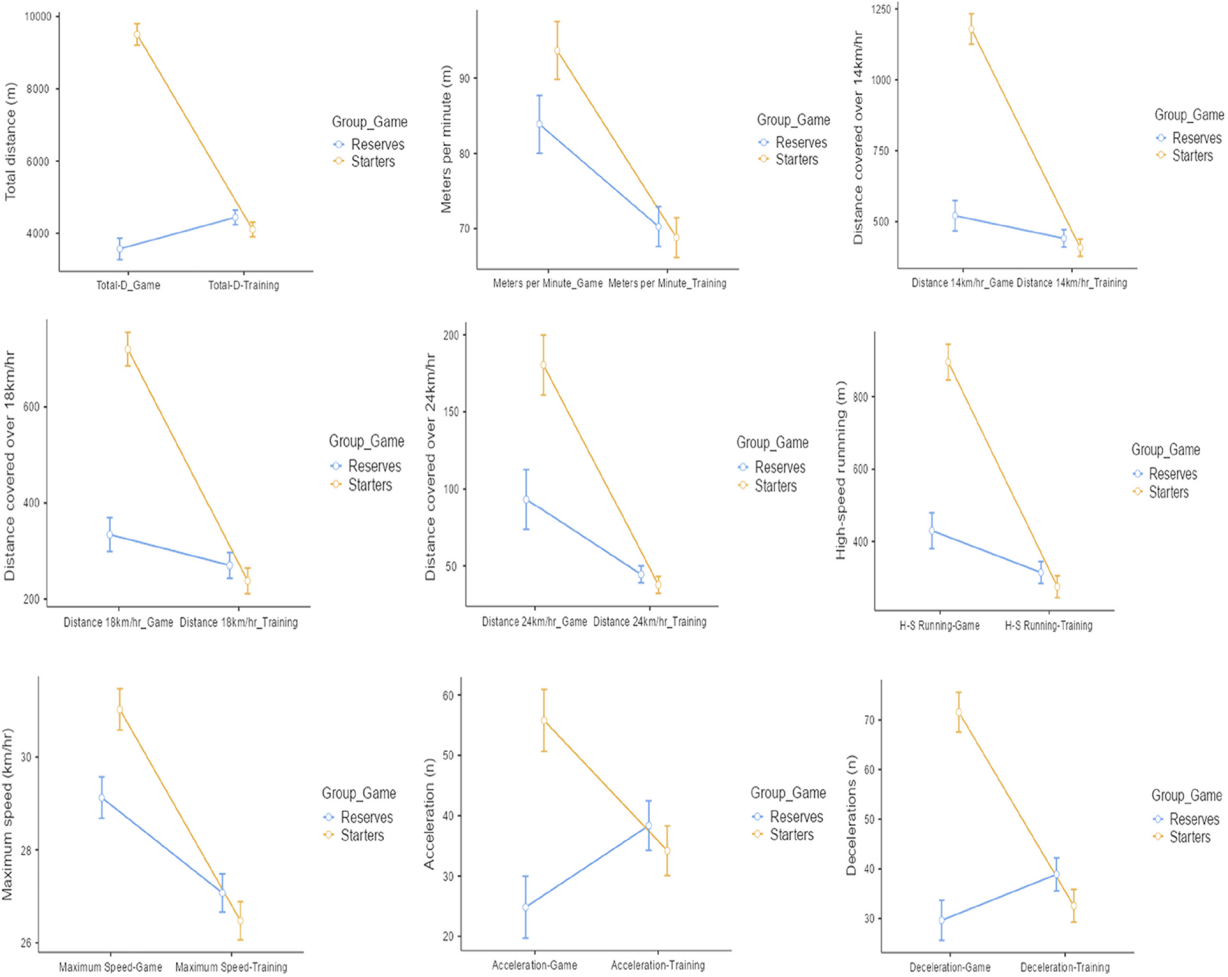
Game and training performance differences on locomotive variables between starters and reserve players.

Comparison between subjects for differences in the training locomotive variables revealed significant differences in 3 variables, namely total distance, maximum speed and decelerations (*p* < 0.05), as demonstrated in Table 2. Reserve players have a comparatively higher performance in all the said parameters, i.e., total distance, maximum speed and decelerations when compared with the starters. Figure 7 illustrates the differences in the players' performances with respect to the training and game locomotive variables.

## 4 Discussion

When analysing the association in the team performances on the games and training locomotive variables, based on coaches' status (i.e., entering or exiting the club), the data showed significant interactions in game vs. training for within-subject and time. With different per cent ranges, it is important to notice that across all analysed variables in competition, the values are superior for the exit coaches, but in training, apart from the total distance, all the other variables presented higher values with the new coaches. Ponce-Bordón et al. (35) research suggested that the HC replacement could positively influence teams' physical performance and match demands, with total distance (in the short term) being significantly greater after the dismissals and other variables getting significantly higher as the season progresses. Compared to our data (related to immediate effect), it is curious to see that total distance is the only variable that does not get higher with the new HC (in this case, in training).

Yet, some differences presented in this data are interesting to acknowledge. The −32,6% (exit HCs) and −26,3% (new HCs) in decelerations achieved in training could be an example of better preparation during the week for the game (values closest to the game demands) from the new HCs, increasing their chances of winning. Same situation with the high-speed running with the old HCs presenting a difference of −59,2% against −51,4% of the new HCs. This data could suggest from the new HCs that it was applied an increased training intensity, more work on tactical behaviours, more liberty to make new adjustments to the teams' playing way and strategies for approaching games, without the need to win immediately, transferring to the game a greater ability to control and balance the team at the different moments of the game, even obtaining a lower physiological performance (25, 36).

In contrast to the current approach, departing HCs might have prompted players to engage in more intense, offensive actions during matches in an effort to secure outcomes that could stave off their potential dismissal. Such strategies could lead to an imbalance within the team, imposing greater physiological demands on the players who may not be adequately conditioned due to the previous training load volumes not supporting such intense efforts. This theory could help explain the findings illustrated in Figure 2, where players showed increased meters covered per minute in games coached by the HCs who were exiting.

Still, towards the game loads not reflecting the training-related performance, it is important to understand the variation of the distribution of the workload during a microcycle span. High-level competition is progressively growing its demands, with the increasingly higher urgency of players to attain optimal physical readiness to withstand the game demands (37, 38). Current research indicates that the higher amount of training load, normally, is concentrated and distributed on the 3rd and 4th days of the week, more oriented towards strength, power and speed training components. Even so, there is a progressive decline until one day before the match because of the introduction of exercises towards the team's technical-tactical organization and strategy for the next game (3, 39). These exercises normally involve a lot of information transmission and break the intensity of the training. As a result of these training adjustments, the average load accumulated during a weekly training cycle is lower than the load players experience in a game. This is consistent with training guidelines that discourage maintaining the same high level of intensity every day of the training microcycle, which should certainly not match the high intensity of game day (40).

Examining the differences between coaches’ status, considering the degree of association in the indicators of locomotor and mechanical performance between training and competition depending on contextual factors, our data did not show significant differences in the player's physical performances with reference to the locations of the games, but revealed 3 locomotive variables showing significance (all 3 in training), differentiating the team when playing against opponents of different strengths. When preparing to play against a superior team, players showed higher performance in meters covered per minute during the week. Conversely, distances covered at speeds above 18 km/h and high-speed running metrics were greater in preparations for facing an inferior team. It is documented that situational variables can significantly affect physical responses and consequently need to be part of the load programming (38). Recent studies showed higher total distance and high-intensity distance in the training weeks after an away match and after playing an inferior team (41). Higher total distance was verified on training-weeks when the next match was against a superior team (42). Guerrero-Calderón et al. (38) showed that the high-speed running was influenced by the strength of the opposition, and the total load was higher within the training week when the forthcoming match was against a superior opponent, with all these studies showing similar results to our data. Intrinsic motivation boosts in players facing superior teams (the opposite could happen with inferior teams), or preparation for a context of less ball possession, greater displacements need to positional organization due to the superior quality of the adversary, can possibly justify these situations related to the intensity of training within the week preparation for the match.

Regarding the difference between game locomotives and the game outcome during each coaching period, no statistically significant differences were observed in the locomotive variables' when the team was winning, losing, or drawing across the two coaching regimes, in training or in competition. About differences considering the game outcome, statistically significant differences were observed between coaches. It is reasonable to anticipate this scenario, as dismissals often occur after a series of games with poor results. Consequently, any points or victories secured after a coaching change could be perceived as an immediate improvement in the team's performance compared to the previous HCs (36).

Research indicates that limited attention has been paid to quantifying and understanding training loads between starters and reserve players, even with the known effects match exposure presents on player workload management, showing that reserve players received only 1/5 of total match exposure during the season (21). Anderson et al. study (43) found that the total distance for reserves during training sessions was significantly higher but did not indicate differences between player roles when analisyng training and match workloads during the total duration of the season. Our results showed no significant differences when looking at the differences in the team performances based on the games and training locomotive variables centred on players' roles under the different coaches' status. Similar results were obtained by Oliveira et al. (44), suggesting that contemporary soccer training methods can adjust loads and make players respond to training stimuli in a homogeneous way.

Normally, reserve players receive less match-specific loading; hence, they could lack physical capacity maintenance during the season when compared with players with more starts, which implicitly will make their physical abilities develop more due to the greater exposure to game time (45). However, in our results, when considering the measured variables for within-subject and time (game vs. training), the data showed different and significant interactions across all the measured variables. The starters presented higher deficits of performance between game and training loads, with none of the variables being balanced across starters, while reserve players showed an increase in performance (total distance, accelerations and decelerations). These higher values of the reserves can be explained through different situations. First, there is a need to exercise them with greater intensities and volumes of loads during the week to compensate for the lack of competition and game stimulus. Another situation to consider, may be the strategy being developed with the starters for the games on some specific weeks, which may require low-intensity technical-tactical behaviours (e.g., low positional blocks on the playing field in a more positional and less physical strategy) to be trained throughout the training sessions intended for offensive and defensive organizations. This situation may require reserve to adopt the opposite stance, to recreate the opponents' behaviours, without great care or concern (as they are not starters) on the part of the coaching staff in controlling the physiological component of these players. Also, the need to manage fatigue on starters, for them to reach the top of their physical capabilities as the game approaches and the week progresses (44). Further work to comprehend these possible impact factors on players' performance in training is clearly warranted.

Yet, looking objectively at the values of the starters, external load variables of volume such as total distance, high-speed running, and decelerations present medium values during training sessions that don't reflect, at least, 50% of the game demands (TD deficit of 56,8%; HSR deficit of 69,2%; DC deficit of 54,6%). These insufficiencies in the values of physiological variables in the preparation of the team during the training microcycles, based on the average values of the demands of the games, increase the probability of the players not being prepared to compete at the highest level and at the top of their abilities, as well as increasing the chances of excessive fatigue and, consequently, an increase in the frequency of injuries. Another factor that could be considered when interpreting the data would be the psychological component of the players. Psychological processes significantly differ in episodes of winning after winning but also can greatly influence performance when losing after losing (46, 47). So, when working under defeats, less positive results, and unsuccessful performances, players could develop less desire and motivation to train at the top of their abilities, decreasing the workload (especially in the analysed weeks of outgoing coaches).

## 5 Study limitations

A possible limitation of this study is the fact that the analysed training weeks were composed of open microcycles, with only one game in the training periodization. In the total 4 weeks of analysis before the dismissals, it may have occurred possible short/closed microcycles, presenting two games per week. When interpreting this data, there's a need to understand that this factor could influence the distribution of the loads, volumes and training intensities, and also the capacity to perform of the players in the weeks analysed. With weeks of closed microcycles, the most utilized players are usually more protected in the few training sessions between games, which could mean that less-used players have a greater capacity to train when open weeks return. This situation could influence the data analysed, by the fact that the team have players with different training and game loads analysed, but with the same training session time performed.

Utilizing two different GPS devices also can be considered a study limitation. The literature states that for most variables analyzed (related with distances and speeds), there is no problem using and comparing both data collection systems. With other ones we need to be careful in the readings and interpretation of the data (like acceleration indices) (48). Also, regarding data collection and interpretation challenges, not having information about the acute and chronic load of the players makes it more difficult to create possible justifications to understand some results obtained, such as the differences observed between starters and reserves, possibly creating confounding factors in some data interpretation.

Additionally, the analysis did not account for technical factors like playing style, team formation systems, and other performance metrics such as ball possession. While the study's focus was on physical performance, it is important to note that existing literature suggests a strong correlation between physical and skill-related performance, with the former potentially impacting the team's tactical execution (49, 50).

## 6 Practical implications and future research

It is proven that contextual factors can significantly alter the performance of players. Most of the studies developed directed their focus to the analysis of the match load, not considering the training load of the training weeks prior to the matches nor associating the influence of factors such as match location, quality of opposition or HCs’ changes (among others) when managing the training load periodization. This type of information might be very useful for HCs and their coaching staff to improve the training load programming. For instance, when facing away games that involve lengthy travel, it's crucial to adjust the volume and intensity of training in the preceding microcycle. Such adjustments help to minimize players’ fatigue accumulation and prevent the compounding effects of travel-related exhaustion with training fatigue. Additionally, the return from these away games may imply specific care at the beginning of the next training microcycle, with load management adapted to players with more playing time.

Furthermore, when they are involved in technical command changes, they need to be aware of these situations of possible disruptions in team dynamics. For future research, it may be interesting to associate physiological performance with the psychological states of players when dismissing and hiring coaches. By collecting data related to well-being and wellness during the beginning of all training sessions and all competitive games, it could be interesting to compare and understand whether there is a possible correlation between low/high performance in training and games resulting from emotional and psychological states of the players, under the command of a HCs who are not getting results, and under a HC who comes to replace the former coach. Also, the need to understanding how certain stimuli are relevant in different game contexts (e.g., knockout games vs. championship games) and associate how they influence physical, mental and tactical behaviours on players performance and preparation, could present an interesting prospect for investigation (51). Another further research avenue could involve qualitative studies, gathering insights from coaches and players who have experienced the mid-season dismissals and appointments of HC to understand the impacts of these changes.

## 7 Conclusions

A common principle among practitioners is that players will compete as they train. With the main physical objective of training being the preparation to withstand the competition demands, insights on the training periodization considering contextual factors could and should be made on high-level soccer teams. Monitoring and quantifying the workload are undoubtedly of increasing importance in professional soccer, targeting the optimization of the player's physical capability based on the specific demands the competition requires. Hence, these contextual variable specificities must be considered by practitioners when managing, developing, and reporting about the team's workload, as our study (quality of the opposition), as well as others, suggests that contextual factors can alter the training load developed by players.

However, tapering strategies by new HCs, or even intern policies at the clubs' dynamics, can influence planning and training periodization. Thus, further studies should try to associate the connection between the training context and clubs' structural/logistical policies to see how the training load interacts with diverse contexts.

## Data Availability

The datasets presented in this article are not readily available Due to an agreement between institutions on protocol conditions for sharing information. Requests to access the datasets should be directed to honoratosousa@hotmail.com.
